# Supplementary material for: Are carbon emissions trading and green financial instruments synergistic? -Comprehensive quantitative research based on content analysis
Source: PLoS One. 2024 Mar 7;19(3):e0298601. doi: 10.1371/journal.pone.0298601 (PMC10919601; doi:10.1371/journal.pone.0298601)
Supplement: S1 Data — (ZIP) [file pone.0298601.s001.zip › Data/地级市Z_F工作报告.zip!/║╙▒▒/║Γ╦«╩╨/└┤╫╘╣1⁄2╓┌║┼í╛┬φ┐╦╩2╛▌═°í┐_2009.docx]

各位代表：

现在，我代表县人民政府向大会作工作报告，请予审议。并请县政协各位委员和列席会议的同志提出意见。

2008年工作回顾

过去的一年，是枣强经受特殊考验、实现转折突破的一年。我们在市委、市政府和县委的正确领导下，在县人大、县政协的监督支持下，团结和带领全县人民，深入落实科学发展观，着力破解障碍发展的深层次矛盾和问题，鼓劲加压，攻坚克难，较好地完成了县十四届人大三次会议确定的各项目标任务。

综合实力稳步增长。全县实现生产总值44.7亿元，同比增长10.6 %；完成财政收入1.77亿元，增长9.9 %；完成全社会固定资产投资17.5亿元，增长36%，其中城镇固定资产投资9.5亿元；农民人均纯收入达到3713元，增长6%。在金融危机的大背景下，全县经济实现了平稳运行。

工业经济蓄势升温。主导产业迈出新步伐。皮毛业研究出台多项扶持政策，采取多种有效措施，引导外迁企业返乡创业，到年底已有80家企业实现“凤还巢”，一般纳税人企业达到90家。成功举办了中国大营第十七届国际皮草交易会和春季裘皮制品订货会，营皮知名度得到新的提升。与乐亭、昌黎、大安、苍山等生皮原产地缔结了友好市县，畅通了购销渠道。我县被中国皮革协会授予“中国皮草科学发展示范基地”，大营镇被中国商业联合会命名为“中国市场名镇”。玻璃钢和机械制造业积极调整产品结构，开发了新型复合管道、热塑汽车部件、掘进机等新产品20多项，产业链条不断延伸，综合竞争力得到新的提升。两大产业纳税分别增长20%和46%。项目建设取得新进展。全年谋划建设300万元以上项目80个，总投资45.7亿元。6个项目列入省重点，枣玻沼气池、驹王专用车、北方农药等一批新项目竣工投产，为经济发展注入了活力。

农业发展扎实推进。国家强农惠农政策得到不折不扣落实，发放各类补贴6469万元。农业生产条件进一步改善，新打机井85眼，铺设防渗管道20.6万米，建成咸淡混浇井组80个，粮食总产量达到32.5万吨，被农业部命名为“全国粮食生产先进县”。积极化解三鹿事件对畜牧业的冲击，新增奶牛2130头，斯格猪出栏8.4万头，特种养殖出栏56万只。全年植树82万株，造林绿化8000亩，被省政府评为“全省农田林网建设先进县”。芍药村裘皮合作社和大王村科技服务合作社初见成效，为农村合作组织建设积累了经验。

城乡面貌明显改观。深入推进“三年大变样”工作，确立了滨河、生态、宜居、和谐的发展定位，启动建设了一批重点工程。当代城市花园三期基本竣工，第四中学建成招生，信誉楼商厦完成主体工程，活动中心部分投入使用，县城功能不断完善。完成枣强新河北段靓化和滨河公园建设，硬化面积2万平米，种植花木20万株，初具城市景观特色。完成了东环路北段拓宽改造、部分小街小巷硬化、亮化和新华东街拆墙透绿，城区新增绿地面积2万平米，绿化覆盖率达到19.3%。对主干街道进行专项治理，规范广告牌匾，县城形象有所改观。郑昔路枣强段加宽、枣景路二期、皮草环线二期建成通车，群众出行更加顺畅。积极推进肖张新民居示范工程， 全县文明生态村达到181 个。

社会事业全面进步。全面落实促进再就业各项扶助政策，新增就业岗位1800个，向外输出劳务8000人次。城乡低保实现动态管理，低保对象由年初的4451人增加到16009人，发放低保金459万元。新增农村五保供养对象823人，全县达到1383人，发放供养金172万元。科技工作成果丰硕，引进新技术、新成果9项，开发省级新产品42项、国家专利12 项。扎实推进义务教育经费保障机制改革，全部免除义务教育阶段学杂费和教科书费，对全县140所中小学实施取暖设施改造。新型农村合作医疗参合率达到91%，累计报免医疗费用1300多万元，受益群众1.5万人次。节能减排取得新成效，县城污水处理厂建成并试运营，单位GDP能耗比上年下降9%，削减COD排量1551 吨、二氧化硫排量426 吨，圆满完成了年度目标任务。积极开展抗震救灾，为四川灾区捐款捐物价值近千万元，完成了125 套过渡安置房的援建任务。妥善处理三鹿奶粉事件，高效完成了专项整治工作。加强社会维稳，圆满完成了奥运安保任务。同时，广播、档案、物价、审计、计生、人民武装等工作都取得了新成绩。

自身建设不断加强。深入推进依法行政，自觉接受县人大的法律监督和县政协的民主监督，主动邀请县人大、县政协领导列席县政府常务会议。办理市县人大代表建议和政协委员提案162件，办复率100%。积极开展“讲党性、知荣辱、做贡献、促发展”活动，不断改进作风，转变职能，取消行政审批项目177项，调整行政审批项目105项，群众满意度不断攀升。

各位代表，过去一年的成绩来之不易，这是县委把握大局、正确领导的结果，是县人大、县政协有效监督、全力支持的结果，是各级干部服务大局、共同努力的结果，是全县38万人民团结奋斗、顽强拼搏的结果。在此，我代表县政府，向全县人民，向各位人大代表、政协委员和各位离退休老同志，向所有关心、支持、参与枣强发展的各界人士和朋友，表示崇高的敬意和衷心的感谢！

在肯定成绩的同时，我们也清醒地看到，我县经济社会发展中还存在一些突出矛盾和问题。主要表现在：县域经济综合实力不强，与先进地区差距越拉越大；主导产业发展水平不高，缺乏大企业、大项目的支撑；农业基础薄弱，产业化水平低，农民增收难度加大；城市功能不够完善，管理机制相对滞后，实现“三年大变样”需付出加倍努力；财政收支矛盾突出，保运转、促发展的压力还比较大；社会事业欠帐较多，安全生产、节能减排还存在不少薄弱环节，促进社会和谐发展任重道远；一些政府部门仍存在责任不强、效能不高、政令不畅、服务不优等现象，发展环境亟待改善。这些问题我们将在今后的工作中采取有效措施认真加以解决。

2009年主要任务

今年，是县委提出“三创一超”（创业富民、创新强县、创优争先，超常发展）、“奋战三年跻身全市先进行列”的首战之年，也是枣强负重奋进、爬坡过坎的关键一年。做好今年的工作意义十分重大。根据县委九届四次全会精神，政府工作的总体要求是：全面贯彻落实科学发展观，以“三创一超”为主线，以项目建设统揽政府工作全局，抓产业促升级，抓城建促变样，抓增收促翻番，抓民生促和谐，全力推进经济社会又好又快发展。主要预期目标是：生产总值增长12%；财政收入增长26.1%；全社会固定资产投资增长35%，其中城镇固定资产投资增长40%；城镇人均可支配收入增长10%；农民人均纯收入增长7.2%。

今年以来，在国际金融危机深入蔓延、经济形势复杂多变的严峻形势下，我们坚持以学习实践科学发展观活动为抓手，引导全县上下坚定信心，迎难而上，开拓进取，促进了经济社会的持续、健康发展。1－5月份，全县实现生产总值21亿元，同比增长12%；完成财政收入8749万元，增长25%；全社会固定资产投资完成6.6亿元，增长45%，其中城镇固定资产投资完成3.5亿元，增长46 %。主要经济指标跟上或超过了时间进度，为完成全年目标任务奠定了基础。纵观当前形势，我们正处在挑战与机遇并存、危机与希望同在的关键时期。尽管金融危机对经济发展带来一定影响，但随着国家一系列扩大内需政策的实施，也给各地上项目、调结构、扩市场带来重大机遇，新一轮区域发展竞赛正在激烈展开。弯道赛跑，既容易迎头赶上，也容易拉大差距。关键取决于我们的精神状态，取决于我们的气魄胆识，取决于我们的工作力度，取决于我们的开拓能力。我们必须审时度势，积极应对，迎难而上，变压力为动力、化挑战为机遇，努力在新一轮发展中抢占先机，赢得主动，推动全县经济社会科学发展、超常发展。

第一，向项目建设要动力，做强超常发展的载体

项目建设牵动经济社会全局，是扩张总量、增强实力，调整结构、提升水平的唯一途径，也是实现超常发展的唯一选择。我们与先进地区的差距就在于没有大项目、好项目，必须咬定项目建设不放松，在大项目、好项目上攻坚突破。

浓厚氛围，营造强大声势。牢固树立“发展为上、项目第一”的意识，把项目建设作为衡量干部能力水平的试金石、有为无为的分水岭。完善项目建设、招商引资奖惩机制，加强调度，严格考核，进一步把各级干部推向项目建设和招商引资主战场，努力形成个个为项目建设尽职责，人人为招商引资做贡献的生动局面，真正实现大项目顶天立地、好项目园区遍地、小项目铺天盖地，所有项目都能真正落地。

放宽视野，力促项目生成。企业内生上项目。鼓励现有企业采取多种形式进行战略重组，加强内引外联，扩大生产规模，加快技术创新，做好项目的谋划与实施。加强跑办争项目。抓住当前有利时机，认真研究国家投资政策，精心谋划一批事关枣强长远的大项目、好项目，全年争列省重点项目10个。强化招商引项目。把引进战略支撑项目作为工作重点，瞄准国内外500强和“中”字头、“国”字号大企业，坚持以政府为主导，积极开展产业招商、园区招商、特色招商，努力取得重大突破。

强力攻坚，破解瓶颈制约。加强重点项目跟进服务，对重大项目落实“一个项目、一套班子、一抓到底”的分包责任制，推行预审、联审、限时、代办、特批工作机制，促进项目顺利实施。科学规划用地指标，充分挖掘土地潜力，确保项目用地。搞好银企对接，营造良好信贷环境，破解资金难题。

分秒必争，推进现有项目。对已谋划建设的52个千万元以上项目，要强化措施，加大推进力度。投资超10亿元的多肽食品工业园要加快前期工作，力促尽早落地。投资6.5亿元的恒润复合管道项目要抓紧落实各种要件，尽快开工建设。京华防腐、豪泰铸造、郁宏搪瓷、达盛昌皮草等已开工项目，要倒排工期，挂图作战，促其早投产、早见效，确保完成“1347”目标任务。

第二，向主导产业要活力，壮大超常发展的支撑

主导产业是枣强经济的命脉，必须竭尽全力、不遗余力地把产业谋划好、发展好。要围绕三年倍增目标，按照新型工业化的要求，加快推进传统产业向现代产业转变，努力提升主导产业在国际国内市场的竞争力、影响力。

提升优势，力促皮毛产业走向新繁荣。税案已经结束，皮毛业进入了全新的发展时期。繁荣皮毛业是人心所向、大势所趋、发展所需，也是当前的头等大事。我们必须因势利导，顺势而为，为营皮振兴打造良好环境，创造有利条件。谋划要有大气魄。要按照“提升规划、提升环境、提升品味”的要求，精心谋划大营镇区建设和产业发展，加快打造“中国裘皮名城”。扶持要有大力度。只要有利于皮毛业科学发展，需要什么政策就给什么政策，需要什么条件就创造什么条件。要大力开展入企帮扶活动，切实解决制约发展的各种问题，为皮毛业发展扫清障碍。旗帜鲜明、大张旗鼓地鼓励引导企业挺直腰杆致富，理直气壮发展。产品要有大提高。大力发展裘皮服装加工，推进产业重心整体上移，实现初级产品集散基地向服装生产基地转型升级。积极引进先进设备、技术，增强研发能力，力求在设计上引领时尚，在工艺上行业领先，实现时尚化、高档化、品牌化。金帝、裘佳怡、瑞裘等6个知名品牌，争列省著名商标。市场要有大发展。加快裘皮展销馆建设，办好一年一度的大营国际皮草交易会，扩大营皮知名度。培育达盛昌皮草公司等外销龙头，积极发展出口贸易，巩固俄罗斯、日韩市场，开发欧美市场；发挥交易中心、红运皮草公司带动作用，努力拓展国内市场。年内一般纳税人企业达到280家，上交税金超过5000万元。

提升水平，推动玻璃钢、机械制造业跃上新台阶。以先进装备促提升。按照“传统产业升级改造、新兴产业高点发展”的思路，鼓励企业瞄准业内领先水平，引进先进设备，提高工艺水平，促进产品向定型化、标准化、品牌化发展。恒润牌管道争创中国驰名商标，彗星牌调压器、黑龙港牌输送机械争创省著名商标。以材料革新促提升。引导玻璃钢企业应用新材料，采用新技术，紧跟科技前沿，大力开发新品，不断提高产品性能和档次。以拓展市场促提升。玻璃钢业巩固管道、沼气池等优势产品，积极开拓航空航天、家居民用新领域。机械制造业适应市场需求，向产品多样化、高档化迈进。以优化结构促提升。引导企业发挥自身优势，做大主导产品，细化分工，延伸链条，促进产业向专业化、精细化、合作化发展。在抓好主导产业升级的同时，积极引进高新技术产业，加快复兴线缆、印刷、化工产业，培育新的经济增长极，努力构建主业突出、多点支撑、梯次发展的产业格局。

提升骨干，实现龙头企业新突破。筛选30家骨干企业，在纳税增长、科技创新、装备提升等方面予以重点扶持，着力培育一批实力雄厚，核心竞争力强， 带动作用明显的产业龙头。引导企业发扬创业、创新、创优精神，推进观念创新、管理创新和机制创新，谋求大发展，力求大突破，加快做大做强。力争到年底，纳税200万元以上的企业达到20家、千万元以上企业实现零的突破。加强工业运行调节，密切关注市场变化，搞好骨干企业、利税大户的动态分析，帮助企业积极应对金融危机，保持稳定经营。

提升平台，打造发展新亮点。重点建设玻璃钢、皮毛两大工业园区。抓住新一轮土地修编机遇，拉大玻璃钢工业园区框架，规划用地面积由现在的3平方公里扩大到5平方公里。加快推进玻璃钢工业园、中小企业创业园的整合提升，统一规划、统一基础设施、统一管理体制、统一推介招商，进一步增强承载能力。启动建设营新工业区，与大营皮毛工业区优势互补、资源共享，进一步拓展发展空间。按照错位发展、适度聚集的要求，加快张新、马屯等工业区建设，为中小项目发展创造条件。借助大广高速机遇，谋划建设城西高新技术产业聚集区，搞好规划设计，加快启动实施。坚持大项目、好项目向县城集中，对入园项目，严把投资规模、投资强度关，坚决杜绝低水平重复建设。

同时，大力开展全民创业，彻底放开政策、放宽领域、放低门槛，打造良好的创业环境，使创业者政治上有荣誉、社会上有地位，让市场主体活起来，多起来，掀起全民创业新热潮。

第三，向城市建设要魅力，创优超常发展的环境

城市既是人居环境，又是投资环境，既是生存需要，又是对外形象。有魅力、有人气的地方也是经济最活跃、发展最有希望的地方。要借助“三年大变样”的东风，把县城建设成独具特色的魅力城市，为经济社会发展搭建良好平台。

着眼长远，切实把城市规划好。按照“滨河、生态、宜居、和谐”的定位，抓住新一轮城市规划修编机遇，坚持大手笔、大气魄，完善规划，拉大框架，到2020 年，城区面积扩大到18平方公里。要把人口发展、产业发展与城市建设统筹推进，加大城中村改造力度，促进村庄向社区转化，农民向市民转化。大力发展城区二、三产业，加快人口聚集，到2020年城市人口发展到18万人。要将城市发展与城西高新技术产业聚集区统筹规划，重点向西、向北发展，主动融入衡水“水市湖城”、“一湖三星”（桃城、冀州、枣强）战略大格局。规划要体现特色，结合枣强实际，把塑造生态特色作为城市远景目标和发展方向。大幅增加绿量，通过实施“一街一品、一路一景”，把县城街道打造成靓丽的风景线。大力度扩大林地面积，让城市走进森林，让森林拥抱城市。力争通过3?5年的努力，把县城建成绿草如茵、花树掩映、城在景中、人在画中的生态名城。

塑造精品，切实把城市建设好。重点抓好“一区二场三网”建设。一区，即枣北新区。坚持高起点规划，高标准建设，吸引有实力、有资质的开发商组团开发，力争今年出进度、明年见成效、后年有形象，把枣北新区打造成三年大变样的示范工程和亮点景区。围绕枣北新区、新河两岸和县城出口，加大拆迁力度，为城市发展腾出空间。二场，即污水处理厂和垃圾处理场。污水处理厂搞好设备调试，完善运营机制，确保通过验收，实现达标排放。规划建设投资4200万元，日处理能力180吨的县城垃圾处理场，年内完成前期准备工作，力争启动主体工程。三网，即城区路网、水网、环城林网。加快平原街、工业街西延和东环路、富强路拓宽改造，打通断头路，形成四通八达、交通便利的城区路网框架。启动枣强新河南段改造，规划建设森林公园人工湖，与枣强新河、索泸河、污水处理厂相互贯通，构建完整畅通的环城水系。环县城建设50米宽的绿化带，形成景色秀丽、生态宜人的城市“绿肺”。全年完成植树9.7万株，绿化12.5公里。同时，精心谋划建设具有枣强特色，能够代表枣强形象的标志性建筑，努力打造城市名片，增加文化内涵。

标本兼治，切实把城市管理好。把城市管理放在更加突出的位置，积极实施划区分级管理机制，明确目标，落实责任，努力实现精细化管理、标准化管理。加大综合执法力度，整顿规范建筑市场，坚决打击乱占、乱建行为，切实维护县城规划的刚性。科学规划集贸市场、社区市场，大力开展环境卫生、马路市场、违规施工、道路交通等专项整治，使县城秩序更加井然、街区更加整洁、交通更加顺畅、环境更加优美。

各乡镇要因地制宜、完善规划、量力推进，加快小城镇建设，力争全县城镇化率提高3个百分点，达到35%。

第四，向农业发展要潜力，夯实超常发展的基础

围绕农业增效、农民增收、农村发展，着力实施农民收入翻番工程，突出做好“三篇文章”，全面推进新农村建设，加快农业大县向农业强县转变。

做好农民增收文章，拓宽增收渠道。靠产业化增收。积极扶持产业化典型，新建规模猪场3个，斯格猪达到1.5万头，奶牛存栏达到3000头、特种养殖达到60万只。全年植树86万株，果品产量达到6.5万吨。组织好无公害农产品认证，新增脱毒甘薯、冷鲜肉制品、肖张果品3个农业品牌。靠科技化增收。深入开展农业科技进村入户活动，加快新技术、新品种、新机具的推广应用，良种覆盖率达到95%以上。做好农作物病虫害、重大气象灾害的预警防御，提高农业增收能力。靠市场化增收。按照“因地制宜、多元创办、政府扶持、市场运作”的思路，大力发展产销衔接、贸工农一体化的专业合作组织，提高农户进入市场的组织化程度。

做好基础建设文章，改善农村环境。完成皮草环线二期、魏广路、营新路、秀贞路等140公里农村公路建设，努力构建主干相通、支线相连、纵横交织、便捷顺畅的交通网络。推广唐林试点经验，大力推进人饮安全供水工程。发展咸淡混浇井组100组，新增节水灌溉面积2万亩。通过向上争取，启动建设覆盖全县的“引黄农灌”工程，确保灌溉用水。新建城北110千伏和石村35千伏变电站，进一步改善电力条件。加快推进新民居建设，每个乡镇启动一个试点村，力争建成一批体现燕赵新民居特色的样板村。

做好农业开放文章，开发农业潜力。抓住国家扶持新农村建设的机遇，加强跑办，争取更多的涉农项目落户枣强。要发挥土地、劳力资源优势，围绕奶牛、生猪、粮食、棉花、林果等产业，强力推介招商，力争年内建成3个投资千万元以上的农产品深加工项目。

同时，要不折不扣地落实上级惠农政策，提高农民种粮积极性。稳定和完善农村土地承包关系，积极规范引导土地向种田能手、龙头企业和农业合作组织流转。推进农村劳动力转移，输出劳务1万人次。

第五，向社会民生要助力，提升超常发展的水平

坚持把改善民生作为政府工作的出发点和落脚点，从群众最关心、最直接、最现实的利益问题入手，加大惠民力度，完善保障机制，不断提高人民群众的幸福指数。

切实做好就业工作。实施更加积极的就业政策，建立健全就业援助机制，着力解决下岗失业人员、零就业家庭、高校毕业生和退伍军人的就业安置。年内提供就业岗位1200个，安置下岗失业人员200人，城镇失业率控制在3%以内，公益性岗位补贴标准由原来的每月300元提高到500元。

不断完善社会保障。进一步扩大社会保险覆盖面，推进非公有制企业和城镇灵活就业人员的参保工作，争取创建国家新型农村养老保险试点县。实施城镇居民医疗保险制度。将破产改制的国有集体企业2000多名退休人员，纳入城镇职工医疗保险。认真做好低保扩面提标工作，实现动态管理下的应保尽保。完成县城中心敬老院和马屯、唐林区域敬老院建设，五保户集中供养率达到50%以上。加快廉租住房建设，努力解决低收入家庭住房问题。

加快社会事业发展。强化科技对经济的支撑作用，增强科技成果推广转化能力，提高科技进步对经济发展的贡献率。切实把教育放在优先发展的地位，确保中小学公用经费、教职工工资按时足额发放。优化教育资源，提高教学质量，高中阶段教育普及率达到65%以上。启动职教中心扩建工程，努力把职教中心建成全省重点职业技术学校。加强卫生设施建设，启动县医院搬迁改造，新建、扩建枣强镇、大营、马屯、唐林、恩察五个乡镇卫生院，努力为群众提供安全、有效、方便、价廉的医疗服务。深化新型农村合作医疗，进一步提高报免比例。严格落实计生惠民政策，稳定低生育水平。积极开展经常性群众文化活动，深入实施电影下乡和送书下乡工程，繁荣农村文化生活。

着力推进节能减排。对新上项目实行严格的节能环评和审查登记，严把环保节能关。加快节能、减排项目建设，对重点排污单位，积极开展专项治理，采取在线监控，实现达标运行。确保全年单位GDP能耗下降10%，削减COD排量2400吨、二氧化硫排量560吨。

全力维护社会稳定。扎实开展安全生产“攻坚年”活动，强化烟花爆竹、危险化学品、人员密集场所、道路交通、建筑施工以及特种设备的安全专项整治。建立责任追究制度，严格行政问责，坚决惩处安全事故的失职、渎职行为。坚持群防群治，加强社会治安综合治理。严厉打击各类刑事犯罪，突出查办涉及民生民计案件。完善应急管理，提高处理突发事件的能力。进一步加强信访工作，健全矛盾纠纷排查调处机制，切实维护社会和谐稳定。

此外，认真做好国防动员、审计、统计、物价、档案、邮政等工作。

第六，向政府建设要效力，强化超常发展的保障

面对艰巨的发展任务，面对人民的重托，加强政府自身建设尤为重要。必须按照为民、务实、清廉、高效的要求，围绕“三创一超”，努力把政府行政能力和服务能力提高到一个新水平。

强化创新意识，转变发展理念。观念支配行动，思路决定出路。能否抓住机遇，实现超常发展，关键取决于我们的思想观念和发展理念能否创新。必须敢于冲破一切有碍于发展的陈腐观念，摒弃一切落后于时代的套路和做法，变革一切影响发展的体制弊端。从枣强实际出发，多思创新之路，多谋创新之举，多办创新之事，多作创新之为，努力开创各项工作新局面。

强化执行意识，提高落实能力。越是发展的特殊时期，越要强调政府执行力和落实力。牢固树立“完成任务，拒绝理由”的工作理念，紧紧盯住具体工作不放，真正做到领导指挥在一线，措施落实在一线，问题解决在一线，成效取得在一线。严格落实重点工作分包责任机制、目标管理机制、督查考核机制，对重要工作任务倒排时间、跟踪问效，确保落实。要强化工作纪律，确保令行禁止，政令畅通，形成干事创业的整体合力。

强化服务意识，优化发展环境。以生产要素进入枣强无障碍为目标，以优化经济发展环境为重点，大力开展“干部作风建设年”活动，严格落实首问负责制、限时办结制，继续清理行政审批事项，简化审批流程。畅通项目建设的“绿色通道”，对效率低下、办事拖拉、故意刁难、吃拿卡要的行为，对干扰和破坏发展环境的行为，一经发现核实，从严查处，绝不姑息，真正在全社会营造亲商、安商、扶商、富商的浓厚氛围。

强化民主意识，加强廉政建设。自觉接受人大及其常委会的法律监督、政协的民主监督和人民群众的社会监督。模范遵守廉洁自律各项规定，认真落实党风廉政建设责任制。深入开展工程招投标、土地出让、产权交易、医药购销等领域专项治理活动，严肃查处各类违法违纪案件。坚持勤俭办事业，严格控制一般性开支，把有限的财力最大限度地用在发展经济和改善民生上。

各位代表，我们深知人民政府的权力是人民赋予的，政府的职责就是为人民服务。要时时刻刻把人民利益放在首位，常怀为民之心，勤思富民之策，践行惠民之举，切实为人民群众办实事、办好事、谋利益、增福祉。今年，要突出办好关系群众利益的10件实事：⑴完成皮草环线二期、魏广路、营新路、秀贞路等140公里的农村公路建设；⑵扎实推进农村人饮安全供水工程；⑶完成东环路、富强路拓宽改造和10条小街小巷亮化；⑷完成枣北新区拆迁、规划，启动组团开发；⑸规划建设森林公园人工湖；⑹建设环城林网，年内完成12.5公里；⑺实施城镇居民医疗保险制度，将破产改制的国有集体企业退休人员，纳入城镇职工医疗保险；⑻完成县城中心敬老院和马屯、唐林区域敬老院建设；⑼新建城北110千伏和石村35千伏变电站；⑽完成职教中心扩建工程。

各位代表！我们面临的任务艰巨而繁重，我们肩负的责任重大而光荣。让我们更加紧密地团结起来，在中共枣强县委的坚强领导下，迎接新挑战，抢抓新机遇，开创新局面，为实现“三创一超”的宏伟目标而努力奋斗！
